# Anti-obesity pharmacotherapy for treatment of pediatric type 2 diabetes: Review of the literature and lessons learned from adults

**DOI:** 10.3389/fendo.2022.1043650

**Published:** 2022-10-27

**Authors:** Megan O. Bensignor, Aaron S. Kelly, Silva Arslanian

**Affiliations:** ^1^ Center for Pediatric Obesity Medicine, Department of Pediatrics, University of Minnesota, Minneapolis, MN, United States; ^2^ Center for Pediatric Research in Obesity and Metabolism, Division of Pediatric Endocrinology and Metabolism, University of Pittsburgh Medical Center (UPMC) Children’s Hospital, University of Pittsburgh, Pittsburgh, PA, United States

**Keywords:** obesity, pediatrics, type 2 diabetes (T2D), anti-obesity pharmacotherapy, GLP-1 RA, phentermine/topiramate combination

## Abstract

Type 2 diabetes mellitus (T2DM) in adolescents is a more rapidly progressive disease, associated with earlier and higher rates of microvascular complications than in adults. As obesity is a significant risk factor for T2DM development and progression, the American Diabetes Association (ADA) recommends anti-obesity medications (AOMs) as adjuvant therapy for adults with both T2DM and overweight/obesity. In adults, the addition of AOMs to a diabetes regimen can improve glycemic control, reduce weight, and decrease anti-diabetes medication use. The ADA recommends considering bariatric surgery for adolescents with T2DM who have a BMI >35 kg/m^2^, but did not mention the use of AOMs in their 2022 updated guidelines. Currently, there are three FDA-approved AOMs available for chronic use in adolescents with obesity. Other medications are used in an “off-label” fashion for appetite suppression and BMI reduction. As additional AOMs are being developed and FDA-approved for the pediatric population, new treatment options with novel mechanisms of action will become available for adolescents with T2DM and obesity. In this review, we will discuss the evidence for the use of AOMs in the treatment of T2DM in adolescents, including lessons learned from the adult T2DM literature.

## Introduction

**Figure 1:**
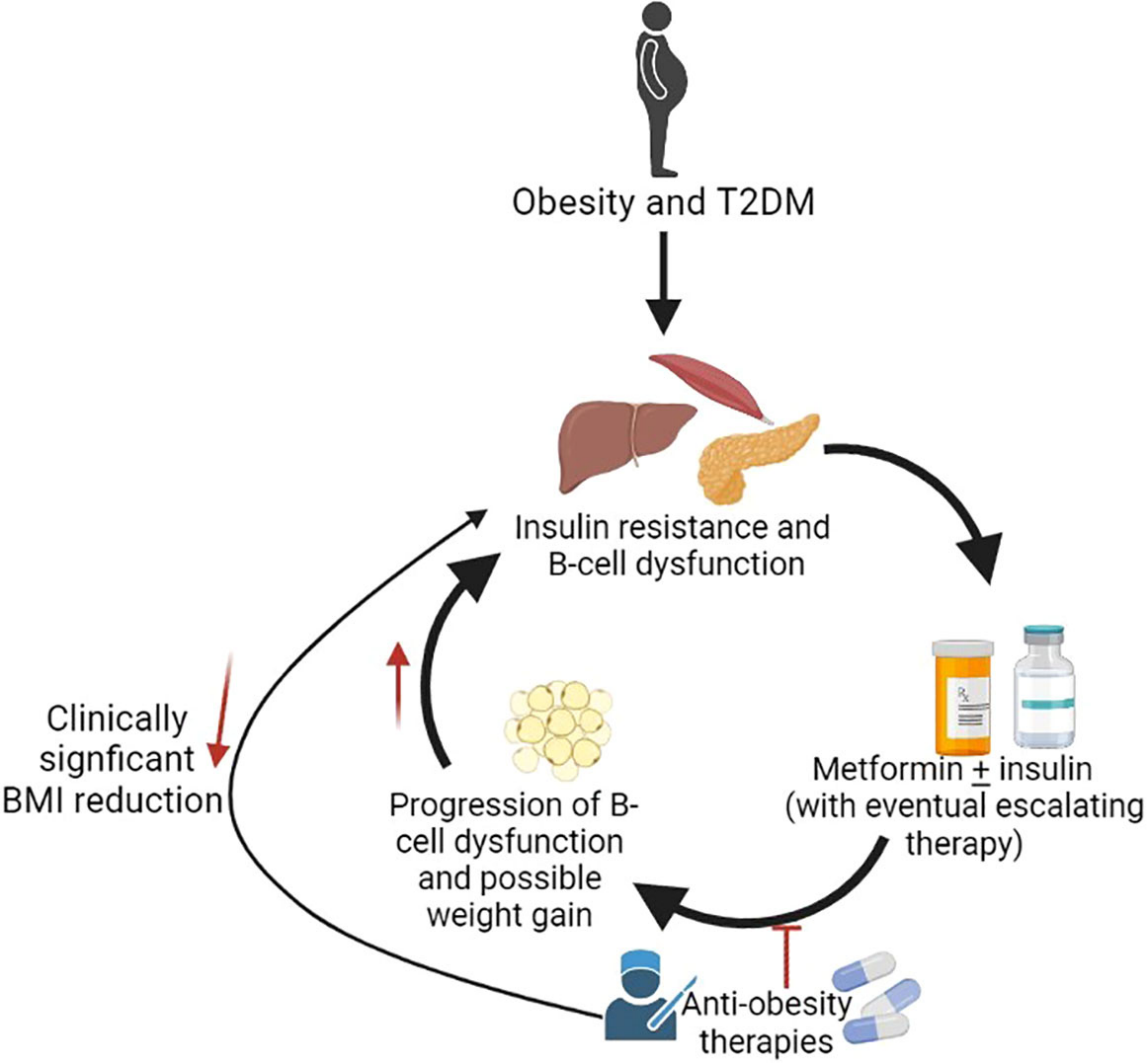
Potential role of anti-obesity medications in treatment of T2DM. Created with BioRender.com.

Having a BMI≥ 95^th^% for age and sex, increases the risk of T2DM (1, 2). Effective obesity management can improve hyperglycemia, reduce anti-diabetes medications, and promote remission in adults with diabetes (3–16). Although a 7-10% weight reduction is recommended by the ADA for youth, the current guidelines recommend lifestyle management and metformin as first-line therapies (17–20), which rarely achieve meaningful BMI reduction or abate T2DM progression in adolescents (21–25). About half of adolescents with T2DM treated with metformin ± lifestyle management progressed to exogenous insulin dependence in a median time of 11.5 months as demonstrated by the Treatment Options for T2DM for Adolescents and Youth (TODAY) study (21). Metformin also did not slow β-cell deterioration in adolescents with impaired glucose tolerance (IGT) or T2DM as shown in the Restoring Insulin Secretion pediatric Study (RISE) (25). Additionally, adults (26, 27) and youth (28) with T2DM often have declining glycemic control over time necessitating increasing insulin requirements, which may be associated with further weight gain (**Figure 1**).

Bariatric surgery is an effective long-term treatment for both T2DM and obesity in adolescents (12, 29). Adolescents who underwent bariatric surgery decreased their Hemoglobin A1c (HbA1c) and BMI after 2 years compared to HbA1c and BMI increases in adolescents medically managed with metformin ± lifestyle management or rosglitazone (12). However, bariatric surgery may not be a feasible option for all patients. As more AOMs become available, there are calls to consider obesity management as the primary goal for T2DM (30). There are currently three FDA-approved AOMs for the chronic treatment of pediatric obesity: phentermine/topiramate (PHN/TPM), liraglutide 3.0 mg/day, and orlistat (26, 31, 32). Semaglutide 2.4 mg and naltrexone/bupropion (NB), which are approved for adults, may be considered for “off-label” use in patients ≥ 16 years with a BMI ≥ 27 kg/m^2^ with T2DM (27). This review article examines the adult and pediatric evidence addressing the potential utility of AOMs to improve glycemic control and elicit BMI reduction in adolescents with obesity and T2DM.

## Anti-obesity medication options

### Phentermine/topiramate

PHN/TPM (Qsymia^®^) is FDA–approved for adolescents ≥ 12 years with a BMI> 95^th^ percentile for age and sex as adjuvant therapy to lifestyle modifications (26). Phentermine and topiramate are thought to 1) reduce appetite through inhibition of norepinephrine reuptake, reduction of hypothalamic glutamate neurotransmission, and lowering neuropeptide Y levels, 2) slow gastric emptying, and 3) increase energy expenditure (28, 33–37). PHN/TPM is a once-daily oral medication (38). The starting dose is 3.75 mg (phentermine) and 23 mg (topiramate) and is up-titrated to effect every 14 days for a maximum dose of 15 mg (phentermine) and 92 mg (topiramate) (38). Topiramate can cause birth defects, and effective contraception should be used in sexually-active patients (38). PHN/TPM is contraindicated in pregnancy, glaucoma, hyperthyroidism, and monoamine oxidase inhibitors (MAOi) use (38). It should be used with caution in those with a history of seizures, renal stones, or depression (39).

Currently, there are no published pediatric trials of PHN/TPM in adolescents with T2DM. However, a randomized placebo-controlled trial (RCT) randomized 223 adolescents (mean age of 14.0 years) with a mean BMI of 37.8 kg/m^2^ 1:1:2 to either placebo, mid-dose PHEN/TPM (7.5 mg/46 mg), and top-dose PHEN/TPM (15 mg/92 mg) (40). The mean BMI reduction was 7.1% for top-dose and 4.8% for mid-dose compared to a gain of 3.3% in the placebo group (40). About 31% of participants receiving mid-dose and 42.5% receiving top-dose PHN/TPM compared to 0% receiving placebo reduced their BMI by ≥ 10% over one year (40). Triglycerides significantly decreased by 12% with both mid-dose and top-dose compared to a 8% increase with placebo (40). This is of interest as excess circulating lipids accumulate in muscle and liver cells, leading to insulin resistance during T2DM pathogenesis (41, 42). However, it is not known if reduced serum triglycerides lead to improved glycemic control as Whole Body Insulin Sensitivity Index did not significantly increase with either dose compared to placebo and no participants with T2DM were enrolled in this trial (40).

The most common side effects reported in this pediatric RCT were headache (placebo: 8.9%, mid-dose: 7.4%, top-dose: 4.4%), nausea (placebo: 3.6%, mid-dose: 3.7%, top-dose: 4.4%), and nasal congestion (placebo: 7.1%, mid-dose: 5.6%, top-dose: 2.7%) (40). Three serious adverse events were reported in two participants randomized to top-dose: suicidal ideation/depression and a bile duct stone (40).

In adults with T2DM and overweight/obesity, two RCTs evaluated the effects of PHN/TPM (**Table 1**). The OB-202/DM-230 trial evaluated top-dose PHN/TPM versus placebo on glycemic control in 130 adults with T2DM controlled with diet or oral medications (6). In this trial, 37% of participants randomized to PHN/TPM versus 9% of the placebo group had ≥10% weight reduction (6). A significantly higher percentage of participants receiving top-dose (32%) achieved an HbA1c < 6.5%. Compared to those receiving placebo (16%) (6). The CONQUER study evaluated mid- and top-dose PHN/TPM versus placebo on change in body weight in adults with a BMI of 27-45 kg/m^2^, including 388 participants with T2DM treated with lifestyle therapy or metformin (7). Participants with T2DM randomized to top- or mid-dose PHN/TPM had a weight reduction of 8.8% and 6.8%, respectively compared to 1.9% in the placebo group (7). Both participants treated with top-and mid-dose had significant reductions in their HbA1c (-0.4 for both doses) compared to placebo (-0.1) (7).

**Table 1:** Anti-obesity pharmacotherapy trials in adults with type 2 diabetes and obesity^a^.

Medication	Study	Study Type	Participant Population	Lifestyle Counseling	HbA1c Δ(percentage points)			Body Weight Δ(percentage)		
					Drug	PbO	P-value	Drug	PbO	P -value
Orlistat 120mg	Hollander et al., 1998 (4)	57 week double-blind RCT	*BMI: 28-40 kg/m^2^ ≥ 18 years * HbA1c: 6.5-10% * Sulfonylurea	* 500 kcal/day deficit	-0.28	+0.18	<0.001	-6.2	-4.3	<0.001
	Berne et al., 2005 (3)	52 week double-blind RCT	* BMI: 28–40 kg/m^2^ * 30–75 years old * HbA1c: 6.5–10% * Metformin ± sulfonylurea	* 600 kcal/day deficit * Increased physical activity	-1.1	−0.2	<0.0001	−5.0	-1.8	<0.0001
Phentermine/topiramate	Gadde et al., 2011 (7)	56 week, double-blind RCT; 2:1:2 randomization to placebo, 7.5/46 or 15/92mg	* BMI: 27-45 kg/m^2^ * 18-70 years * >2 comorbidities * Metformin or diet	* 500 kcal/day deficit	7.5/46 mg: -0.4	-0.1	0.0288	7.5/46 mg: -6.8	-1.9	<0·0001
					15/92 mg: -0.4		0.0043	15/92 mg: -8.8		<0·0001
	Garvey et al., 2014 (6)	56-week, double-blind, RCT	* BMI: 27–45 kg/m^2^ * 18 - 70 years old * HbA1c: 7.0–12.0% * ± oral anti-diabetes drugs	* 500 kcal/day deficit	−1.6	−1.2	< 0.05	-9.6	-2.6	<0.0001
Naltrexone/Bupropion 32/360 mg	Hollander et al., 2013 (5)	56-week, double-blind, RCT; 2:1 randomization to NB or placebo.	* BMI: 27-45 kg/m^2^ * 18–70 years * HbA1c: 7-10% * ± oral anti-diabetes drugs	* 500 kcal/day deficit * Increased physical activity	-0.6	-0.1	<0.001	-5.0	-1.8	<0.001
Liraglutide	Davies et al., 2015 (8)	56 week double-blind RCT; 2:1:1 randomization to 3.0 or 1.8mg/day, or placebo	* BMI: ≥27 kg/m^2^ * ≥ 18 years * HbA1c: 7-10% * ≥ 3 oral anti-diabetes drugs	* 500 kcal/day deficit * Increased physical activity	1.8 mg: -1.1	-0.3	<0.001	1.8 mg -4.7	-2.0	<0.001
					3.0 mg: -1.3		<0.001	3.0 mg: -6.0		<0.001
Semaglutide	Davies et al., 2021 (9)	68 week, double-blind, RCT; 1:1:1: randomization to 2.4 or 1.0 mg, or placebo	* BMI ≥ 27 kg/m^2^ * ≥ 18 years * HbA1c: 7-10% * T2DM dx ≥ 6 months * ≥ 3 oral anti-diabetes drugs	* 500 kcal/day deficit * Increased physical activity	1.0 mg: -1.5	-0.4	0·0001	1.0 mg: -7.0	-3.4	<0·0001
					2.4 mg: -1.6		0·0001	2.4 mg: -9.6		<0·0001

a Trials described are limited to studies discussed in this article.

BMI, body mass index; NB, Naltrexone/Bupropion; PbO, Placebo; RCT, randomized placebo-controlled trial.

The most common adverse events in the PHN/TPM groups in either of the adult trials differed from the pediatric trial, and were paresthesia, (placebo: 3.8%; mid-dose: 7.5%; top-dose: 17.7%), constipation (placebo: 6.4%; mid-dose: 14.9%; top-dose: 7.7%), and insomnia (placebo: 5.1%; mid-dose: 7.5%; top-dose: 14.0%) in the CONQUER trial with similar adverse rates in the OB-202/DM-230 trial (6). A combination of sixty-four hypoglycemic events without severe hypoglycemia occurred for both trials, often with concomitant anti-diabetes medications (6).

It remains to be determined if in youth with T2DM, the results of a PHN/TPM trial will mirror those of adults with T2DM. Until then, PHN/TPM may be considered as a potentially useful adjuvant therapy in adolescents, aged ≥ 12 years, with T2DM who also have obesity.

### Glucagon-like peptide-1 receptor agonists

There are two injectable glucagon-like peptide-1 receptor agonists (GLP-1RAs) that are FDA-approved for youth ≥ 10 years with T2DM: daily liraglutide 1.8 mg/day (Victoza^®^) and once-weekly exenatide extended-release (ER; Bydureon BCise^®^). GLP-1RAs stimulate insulin secretion, decrease glucagon concentration, delay gastric emptying, and decrease appetite, making them an attractive therapy for both obesity and T2DM (43). A history of pancreatitis and personal/family history of medullary thyroid carcinoma or Multiple Endocrine Neoplasia type 2 are contraindications to GLP-1RAs use (44). Liraglutide 1.8 mg/day and exenatide ER are not considered AOMs and may not result in clinically significant BMI reduction. Exenatide ER did not significantly reduce weight compared to placebo in 84 adolescents with T2DM, previously treated with diet and exercise alone or with metformin ± a sulfonylurea and/or insulin after 24 and 52 weeks (45, 46). In terms of liraglutide, 134 adolescents with T2DM and overweight/obesity treated with doses up to 1.8 mg/day did not have significant BMI reduction after a 26-week double-blind RCT (47, 48). Despite the minimal BMI reduction of 0.92 kg/m^2^ at the end of the 26-week open-label extension of this RCT, the percentage of participants receiving basal insulin from baseline to trial end did not change with liraglutide treatment while it significantly increased in the placebo group (48). However, this statistically significant BMI reduction with liraglutide 1.8 mg does not reach the 7-10% weight reduction recommended in ADA guidelines and may not be clinically significant (47, 48).

#### Liraglutide 3.0mg/day

A higher dose of liraglutide at 3.0 mg/day (Saxenda^®^) is FDA-approved for obesity in youth ≥ 12 years, weighing ≥ 60 kg or an initial BMI ≥ 30 kg/m (32). The Satiety and Clinical Adipose-Liraglutide Evidence trial in adolescents with and without T2DM clinic trial (SCALE-TEENs) Trial was a double-blind, placebo-controlled RCT, which randomized 251 participants, aged 12 to <18 years to either liraglutide 3.0 mg/day (mean BMI: 35.3 ± 5.1 kg/m^2^) or placebo (mean BMI: 35.8 ± 5.7 kg/m^2^) (49). Twenty-six percent of participants randomized to liraglutide reduced their BMI by ≥ 10% compared to 8.1% randomized to placebo (49). Mean HbA1c did not change significantly in either the liraglutide or the placebo groups. Only two adolescents with T2DM were enrolled in this trial, hence no inferences could be made concerning BMI reduction and improvement in HbA1C in youth with T2DM on this dose of liraglutide (49).

The most commonly reported adverse events were nausea (placebo: 14.3%; liraglutide: 42.4%), vomiting (placebo: 4.0%; liraglutide: 34.4%), headache (placebo: 27.8%; liraglutide: 23.2%) and diarrhea (placebo: 14.3%; liraglutide: 22.4%) (8, 49). Gastrointestinal side effects mainly occurred during dose escalation (the initial 4 to 8 weeks of treatment) (49). Within the liraglutide group, one participant died by suicide and there was one pancreatitis episode (49). The liraglutide group more commonly experienced hypoglycemia compared to placebo (26% versus 18%) with no severe hypoglycemia (49).

The SCALE-adult study randomized participants with T2DM to either liraglutide 3.0 mg, liraglutide 1.8 mg, or placebo in a 2:1:1 fashion (**Table 1**) (8). Participants randomized to liraglutide 3.0 mg reduced their body weight by 6.0% compared to 4.7% with 1.8 mg and 2.0% with placebo (8). About 25% and 16% of participants in the liraglutide 3.0 mg and 1.8 mg groups, respectively, had a ≥ 10% weight reduction compared to 6.7% in the placebo group (8). HbA1c decreased with liraglutide 3.0 mg by 1.3 percentage points compared to 1.1 percentage points with 1.8 mg and 0.3 percentage points with placebo (8). Additionally, more participants treated with liraglutide at both doses were able to reduce oral anti-diabetes medications than those on placebo (odds ratio: 3.36 with 1.8mg and 5.63 with 3.0mg) (8). The most commonly reported adverse events were nausea, vomiting, and diarrhea, occurring in 65.2% with liraglutide 3.0 mg and 56.2% with liraglutide 1.8 mg (versus 39.2% in placebo) (8). No cases of pancreatitis were reported (8). Hypoglycemia was greatest with liraglutide 3.0 mg (44.5%) compared to placebo (27.4%) or liraglutide 1.8mg (39.4%); there was no severe hypoglycemia in participants who were not concurrently treated with sulfonylureas (8).

Although the 3.0 mg liraglutide dose is FDA-approved for pediatric obesity only, almost all adolescents with T2DM in North America have overweight/obesity and could potentially meet prescribing criteria (50). Pending a trial of 3.0 mg liraglutide in adolescent with T2DM, it is tempting to postulate that results would be similar to that of adult trials of T2DM, resulting in weight and BMI reductions and hyperglycemia improvement.

#### Semaglutide

Semaglutide in its once-weekly injectable GLP-1RA form is approved for adults with a BMI ≥ 30 kg/m^2^ or ≥ 27 kg/m^2^ with ≥ one weight-related comorbidity at doses up to 2.4 mg weekly (Wegovy^®^) (51). Weekly semaglutide injection up to 2.0 mg (Ozempic^®^) is also approved for adults with T2DM (51). Semaglutide at any dose is not yet FDA-approved for any pediatric population, and data from the double-blind, placebo-controlled RCT of semaglutide 2.4 mg in adolescents ≥ 12 years with obesity has not yet been published (52).

In an adult T2DM population, a RCT evaluating the effects of semaglutide 2.4 mg or 1.0 mg versus placebo found that the HbA1c decreased significantly in semaglutide 2.4 mg by 1.6 percentage points versus 1.5 percentage points for 1.0 mg and 0.4 percentage points with placebo (**Table 1**) (9). Concomitant anti-diabetes medications were reduced or discontinued more often in participants receiving 2.4 mg (28.6%) compared to 1.0 mg (25.1%) and placebo (7.1%) (9). More patients (45.6%) receiving 2.4 mg had a ≥ 10% weight reduction than 1.0 mg (28.7%) and placebo (8.2%) (9). Similar to liraglutide, nausea, vomiting, diarrhea, and constipation were the most common adverse events (9). There were two episodes of pancreatitis in the 2.4 mg group and one in the placebo group (9). Severe hypoglycemia events were also more common with 2.4 mg (5.7%) and 1.0 mg (5.5%) compared with placebo (3.0%) (9).

Semaglutide at its anti-obesity dose may be effective adjuvant therapy for adolescents with both T2DM and obesity. Semaglutide could be considered for “off-label” use in adolescents who have not had significant BMI reduction or glycemic control with liraglutide 3.0 mg/day given the superior weight reduction effects of semaglutide 2.4 mg versus liraglutide 3.0 mg in adults with overweight/obesity without diabetes (53). The once-a-week administration may also decrease the burden of diabetes care and improve medication adherence. However, semaglutide is not yet FDA-approved for patients under age 18 years, and safety and efficacy data in a pediatric population has yet to be published at this time.

### Orlistat

Orlistat, which is FDA approved for adolescents ≥ 12 years and adults for obesity, is an oral gastrointestinal lipase inhibitor and is available in over-the-counter (60 mg; Alli^®^) and prescription (120 mg) forms (54). It can be taken up to three times a day with meals and is contraindicated in pregnancy and a history of cholestasis or malabsorption (31).

In an open-label study of twenty adolescents with obesity (mean BMI of 44.1 ± 12.5 kg/m^2^), which included one participant with T2DM and one participant with IGT, Orlistat 120 mg plus behavioral modification reduced body weight by 3.5 ± 6.0% (55). Indices of insulin sensitivity significantly improved after six months of treatment (55). A larger double-blind RCT found that adolescents with obesity receiving orlistat 120 mg reduced their BMI by 0.55 kg/m^2^ compared to an increase of 0.31 kg/m^2^ with placebo (56). However this trial did not include adolescents with T2DM on anti-diabetes medications (56). For both studies, medication adherence was reported as > 70% with fatty/oily stools, increased flatulence, and increased fecal frequency as the main adverse events (55, 56).

Two RCTs found that adult T2DM participants receiving orlistat 120 mg in addition to metformin ± sulfonylurea therapy had greater weight reduction compared to those receiving placebo (**Table 1**) (3, 4). In both trials, orlistat was associated with significant improvement in glycemic control compared to placebo (3, 4). Fecal urgency, fatty/oily stool, fecal incontinence, and increased defecation were almost exclusively related to orlistat (4).

There have been no randomized control trials of orlistat in adolescents with T2DM on anti-diabetes medications. The need for orlistat to be taken with food up to three times a day makes it a less attractive FDA-approved option in an adolescent population, especially considering its gastrointestinal side effects. Lastly, it appears that liraglutide and PHN/TPM result in greater BMI reductions than orlistat when compared to placebo in adolescents with obesity, even though these are not head-to-head trials.

### Naltrexone/bupropion

NB (Contrave^®^), is FDA-approved for chronic weight management in adults (57). In combination, naltrexone and bupropion act on the central nervous system to mediate appetite-suppression and decrease reward-seeking behaviors, reducing food intake (58–60). There is a black box warning for suicidal behavior or ideation in patients ≤ 24 years old with depression (57). NB is contraindicated in pregnancy, uncontrolled hypertension, seizure disorders, glaucoma, opioid use/dependence, eating disorders, or MAOi use (57). There have not been any published pediatric obesity or T2DM studies.

In adults with overweight/obesity and T2DM, a double-blind RCT evaluating the safety and efficacy of NB versus placebo demonstrated significantly greater weight reduction with NB compared to placebo (-5.0% versus -1.8%; **Table 1**) (5). Additionally, more participants treated with NB had an HbA1c of ≤7.0% compared to placebo (44.1% versus 26.3%) (5). The reported side effects, more prevalent in the NB-treated group, were nausea, vomiting, constipation, diarrhea, and headache (5). Depression, suicidal ideation, or hypoglycemia incidence did not differ between the NB and placebo groups (5).

Although NB has been found to reduce BMI and improve glycemic control in adults with T2DM and overweight/obesity, this medication should be used with caution in adolescents, as NB has not been studied in a pediatric population. Furthermore, NB carries a black box warning for suicidal behavior in patients <24 years with depression, a common comorbidity for adolescents with both obesity and diabetes (61–63).

## Discussion

This review of AOMs in T2DM management demonstrates the paucity of RCTs in adolescents with obesity and T2DM, even though there is a growing number of trials in adults (**Table 1**). The evidence for using AOMS in pediatric T2DM treatment include the finding that weight reduction over the first year of T2DM diagnosis correlated with improvement in HbA1c in pediatric patients (64, 65). In terms of youth with obesity and insulin resistance (without diabetes), a BMI reduction of about 8% was the threshold, which improved insulin sensitivity was observed (8). Metformin and lifestyle modifications on average do not result in such BMI reduction in pediatric patients nor do these first-line diabetes therapies prevent long-term exogenous insulin dependence or β-cell dysfunction (66, 67).

However, caution should be taken in applying adult T2DM data to an adolescent population. The lack of pediatric data, including long-term safety and efficacy data, is a drawback of using AOMS in pediatric T2DM treatment. As adolescents with T2DM have hyper-responsive β-cells, lower insulin sensitivity, and more rapid deterioration in β-cell function compared to adults, there may be different effects on glycemic control between adults and adolescents even with similar weight reduction (21, 25). Weight reduction also differs between adults and adolescents with obesity as demonstrated by the adult and pediatric liraglutide data presented. Side effects profiles also may differ between adult and pediatric patients as illustrated by the PHN/TPM data. Additionally, none of the adult T2DM trials included participants on insulin therapy. As adolescents with T2DM become insulin-dependent early in the course of their disease, the exclusion of participants treated with insulin sharply limits the applicability of these studies to adolescents.

In conclusion, the effectiveness of AOMS differs in adolescents with obesity compared with adults, which necessitates RCTs specifically in pediatric T2DM population before they are incorporated into pediatric T2DM guidelines. However, adult T2DM studies suggest that ≥7% weight reduction may be possible with at least three AOMs, two of which are currently FDA-approved for pediatric obesity, but not T2DM. The adult literature demonstrates that weight reduction can improve glycemic control and decrease the number of anti-diabetes medications, and AOMs are incorporated into the adult ADA T2DM guidelines (68). In contrast, the current pediatric guideline-recommend first-line treatments of lifestyle management and metformin rarely result in BMI reduction or prevent disease progression in adolescents (21–25). As many adolescents with T2DM who are severely insulin resistant also have obesity, AOMS may be potentially useful adjuvant therapy to anti-diabetes therapies in those who meet prescribing criteria to improve insulin resistance consequent to weight/BMI reduction, and possibly improve glycemic control.

## Author contributions

MB conducted the literature review. MB, AK, and SA contributed to the writing and manuscript preparation. All authors contributed to the article and approved the submitted version.

## Conflict of interest

AK engages in unpaid consulting and educational activities for Novo Nordisk, Vivus, Eli Lilly, and Boehringer Ingelheim as well as receives donated drug/placebo from Vivus and Novo Nordisk for National Institute of Diabetes and Digestive and Kidney Diseases-funded clinical trials. SA receives grant support from and serves as a consultant for AZ DMC, Novo and Lilly. MB receives research support Vivus Inc and Novo Nordisk.

## Publisher’s note

All claims expressed in this article are solely those of the authors and do not necessarily represent those of their affiliated organizations, or those of the publisher, the editors and the reviewers. Any product that may be evaluated in this article, or claim that may be made by its manufacturer, is not guaranteed or endorsed by the publisher.
